# Interrelationships and Continuities in Symptoms of Oppositional Defiant and Conduct Disorders from Age 4 to 10 in the Community

**DOI:** 10.1007/s10802-016-0210-4

**Published:** 2016-10-25

**Authors:** Silje Merethe Husby, Lars Wichstrøm

**Affiliations:** 1 Department of Psychology, Norwegian University of Science and Technology, Trondheim, Norway; 2 Department of Child and Adolescent Psychiatry, St. Olavs Hospital, Trondheim, Norway; 3 NTNU Social Science, Trondheim, Norway

**Keywords:** Oppositional defiant disorder, Conduct disorder, Longitudinal, Diagnostic interview, Early onset

## Abstract

Childhood oppositional defiant disorder (ODD) has commonly been thought to increase the risk of conduct disorder (CD) in late childhood and adolescence. However, symptoms of CD may also emerge during preschool and middle childhood. The few studies that have examined whether ODD increases the risk of such early onset CD have produced equivocal results, potentially due to methodological issues. In this study, a community sample of Norwegian 4-year-olds (*n* = 1042, 49.9 % males) was examined bi-annually over four waves of data collection. Symptoms of ODD, CD, attention-deficit/hyperactivity disorder (ADHD), anxiety and depressive disorders were measured through interviews with parents and children using the Preschool Age Psychiatric Assessment and the Child and Adolescent Psychiatric Assessment. The results showed that at all ages, more symptoms of ODD predicted more symptoms of CD at the next age of examination even after adjusting for previous CD and comorbid conditions. The effect of previous ODD on CD two years later did not differ according to gender, SES, or parental cohabitating status at any point in time. There was modest homotypical continuity in symptoms of CD and moderate homotypical continuity in symptoms of ODD. Symptoms of ODD increased from age 4 to 8 and declined to age 10. In conclusion, symptoms of ODD increase the risk of early onset symptoms of CD. The continuity in symptoms of ODD, and to some extent CD, combined with an increased risk of early symptoms of CD forecasted by symptoms of ODD, underscore the importance of detection, prevention and treatment of behavioral disorders already in early childhood.

## ᅟ

Behavioral disorders, (oppositional defiant disorder (ODD) and conduct disorder (CD)), are common and prime reasons for referral to child and adolescent psychiatry (Wichstrøm et al. 2014). ODD in childhood has generally been considered a developmental precursor to CD in later childhood and adolescence (e.g., Burke et al. 2002; Loeber and Burke 2011; Moffitt et al. 2008). However, over the last decade, it has become increasingly evident that CD can also be present in the preschool and early school years (e.g., Keenan et al. 2011, 2007; Kim-Cohen et al. 2005). Not all children with ODD progress to CD, and most likely, the progression from ODD to CD is moderated by factors intrinsic to the child or the child’s environment (Holmes et al. 2001). However, before one embarks upon the quest of uncovering which mechanisms cause some but not other young children with ODD to develop CD it is important to determine whether this transition actually occurs at a young age.

Most research regarding the relationship between ODD and CD has been conducted on school-aged children and adolescents (i.e., Burke et al. 2002; Costello et al. 2003; Lahey et al. 2002; Loeber et al. 2000; Rowe et al. 2002). Given the differential developmental levels and differing social contexts of preschoolers and early school-aged children compared to those in late childhood and adolescence, it is possible that the ODD➔CD relationship is different in early childhood as opposed to later in development. To capture most of the developmental transitions between ODD and CD, prospective studies starting at an early age are needed (Burke et al. 2010). Due to several issues of measurement, (i.e., diagnostic assignments, sample type, and accounting for comorbidity), which will be reviewed below, no clear conclusion regarding the ODD/early onset CD relationship can be drawn from existing research. Thus, the primary goal of the current study was to investigate whether symptoms of ODD increase the risk of subsequent symptoms of CD while considering a range of methodological challenges.

### Checklists Versus Interviews

The few existing prospective studies that investigated the ODD-CD relationship in young children can be classified into two broad groups: investigations based on rating scales and studies using diagnostic interviews. The use of ratings scales administered to parents and teachers has made significant contributions to research on disruptive behaviors in children (Egger et al. 2006), but such parent and teacher reports are not without their limitations. First, many rating scales do not provide items that directly correspond to symptoms as defined by the Diagnostic and Statistical Manual of Mental Disorders (DSM). Second, respondents, specifically parents, usually have limited exposure to the full range of normative behaviors that can be displayed by young children. This lack of knowledge might be particularly relevant in the case of ODD and CD (Bufferd et al. 2011) because most young children occasionally engage in behaviors that are disruptive, and parents might have difficulty distinguishing clinically significant symptoms from normative and transient behaviors (Bufferd et al. 2012). This implies that interviewer-based diagnostic interviews (where the interviewer decides whether a symptom is present or absent), which tap specifically into the symptoms of ODD and CD might be particularly well suited for elucidating the relationship between these two disorders.

### Clinical Versus Community Samples

Some studies that applied diagnostic interviews have provided evidence that ODD predicts early onset CD (Burke et al. 2010; Lahey et al. 2009), whereas others have not (Keenan et al. 2011; Rolon-Arroyo et al. 2014; Speltz et al. 1999). Importantly, these investigations examined children who were referred to psychiatric clinics due to disruptive behavior problems or attention-deficit/hyperactivity disorder; ADHD (Burke and Loeber 2010; Keenan et al. 2011; Speltz et al. 1999) or children with pre-existing externalizing problems (Rolon-Arroyo et al. 2014). This is significant, as referred children may present more comorbid conditions than community children (Waschbusch 2002); furthermore, the developmental course of problems across clinically referred cases and non-referred children may prove different (Lahey et al. 1998; Waschbusch 2002). Thus, findings from studies of referred or high-risk children may not be generalizable to the broader population. Those who have examined community-based samples of non-referred children have done so using parent-completed checklists (Diamantopoulou et al. 2011; Lahey et al. 2009). Therefore, we know little about the potential effect of DSM-defined ODD on the subsequent early onset symptoms of CD among community children. However, as seminally chronicled by Moffitt and Caspi (2001) as well as Vaughn et al. (2011), certain children might be on a developmental trajectory toward more serious conduct problems (i.e., CD) because of the severity of their initial problems or for biological or temperamental reasons. Thus, the overall findings from community studies might be driven by a subset of children: those with the most severe problems at the outset. Therefore, we will address the possibility of such non-linear effects.

### Categorical Versus Continuous Parameterization

Studies have differed in their practices concerning diagnostic assignments. When studying non-clinical samples in early childhood, the prevalence of CD is expected to be low. If samples are not very large, they will likely lack the statistical power to detect an ODD➔CD relationship. Even when samples sizes are fairly high, such as in the present study, symptom counts of ODD and CD may be the method of choice in countries where prevalences are low. In addition, CD may develop in a manner where the full range of symptoms is not present until late childhood or adolescence (Fergusson and Horwood 1995). An assessment of symptoms on a continuum may therefore prevent the loss of information that might be discarded when using diagnostic cut-offs, and this approach may be particularly useful for preschoolers (Keenan et al. 2011; Rolon-Arroyo et al. 2014) and early school-aged children. Because subclinical levels of CD in preschoolers are predictive of later CD diagnosis (Keenan et al. 2011), and because we examine this condition in Norway where the prevalence of the disorder is low (Wichstrøm et al. 2012), a continuous approach is appropriate.

### Accounting for Comorbidity

A substantial comorbidity exists between disruptive disorders and other disorders (Lahey et al. 2002) among young children (Wichstrøm et al. 2012). Indeed, there are strong indications that ADHD contributes to the progression from ODD to CD (Diamantopoulou et al. 2011; Waschbusch 2002). Emotional disorders have also been found to co-exist with ODD and CD (Lahey et al. 2002). Studies on older children have shown how controlling for comorbidity can render ODD insignificant in the prediction of later CD (Costello et al. 2003). This indicates the importance of controlling for comorbid conditions when examining the ODD-CD relationship.

### The Stability of ODD and CD Symptoms

The issue of heterotypical continuity between ODD and later CD in early childhood notwithstanding, one would expect homotypical continuity to be present, as indicated by some comparatively short-term prospective research using diagnostic interviews among referred children (Bunte et al. 2014; Keenan et al. 2011) and community children, reporting on ODD but not CD. (Bufferd et al. 2012). Studies using parent-completed questionnaires among community children also identify high heterotypical continuity (Kim-Cohen et al. 2005, 2009), as did a shorter-term study that examined children from preschool to seven years of age using rating scales and an interview (Lavigne et al. 2001). To what extent DSM-defined symptoms of ODD and CD among preschoolers in the community evidence longer-term homotypical continuity into middle childhood is unknown, but will be reported here, as will changes in the mean levels of symptoms over time.

Moreover, some studies have shown ODD to be a stronger predictor of subsequent CD among boys than girls (Rowe et al. 2002), although the findings are conflicting (e.g. Diamantopoulou et al. 2011; Rolon-Arroyo et al. 2014). Therefore, we will conduct gender-specific analyses. Similarly, ODD and CD are profoundly socially stratified, also among young Norwegian children (Wichstrøm et al. 2012), with higher rates among those of low socioeconomic status (SES) and those whose parents do not live together. Economic hardships at both family and neighborhood level are linked to antisocial behavior (Duncan et al. 1994), and boys who live in low-SES neighborhoods might not exhibit the normative decline of antisocial behavior that is expected across childhood. Rather, they might be at risk for following an early-onset, life-course persistent pathway of antisocial behavior (Odgers et al. 2012). Similarly, Loeber et al. (1995) found that low SES, together with oppositional defiant behavior, might predict preadolescent boys’ transition to CD. These findings suggest that stability in symptoms and transitions from ODD to CD are stronger among less privileged children. Therefore, we will examine whether SES and marital status moderate such stabilities and transitions.

### The Current Study

Whether symptoms of ODD increase the probability of developing symptoms of CD in early childhood among community children has yet to be determined. We extend existing research on the ODD➔CD linkage by studying a representative community sample, using an interviewer-based diagnostic interview and starting at age 4 years and spanning a 6-year period. Specifically, we evaluate whether DSM-IV-defined symptoms of ODD increase the probability of future symptoms of CD, while adjusting for concurrent symptoms of CD, ADHD, anxiety and depressive disorders. Moreover, this study represents the first attempt to address whether the DSM-defined symptoms of ODD and CD in preschool continue into middle childhood, whether changes are present in the overall symptom load, and whether gender, SES and marital status differences exist with regard to the ODD-CD relationship. Given the existing literature we tentatively hypothesize that higher number of symptoms of ODD will increase the probability of symptoms of CD two years later, and that symptoms counts of ODD and CD will evidence some stability.

## Methods

### Participants and Procedure

The Trondheim Early Secure Study (TESS) consists of members of the 2003 and 2004 birth cohorts in Trondheim, Norway (Wichstrøm et al. 2012). A letter of invitation together with the Strengths and Difficulties Questionnaire (SDQ) 4–16 version (Crone et al. 2008), a screening assessment for emotional and behavioral problems, was sent to all children in the two birth cohorts’ homes. Parents brought the completed SDQ when they attended the well-child clinic for the routine health check at age 4 years. Most of the informants (84.4 %) were the biological mothers, and the parents were mostly of Norwegian (93.0 %) or Western (2.7 %) origin. The sample consisted predominantly of married (56.3 %) or cohabiting couples (32.6 %). All parents had completed compulsory education,( i.e., the 9th grade), and 58.3 % had a college degree or higher. The annual gross family income was above $107,000 (USD) in 26.7 % of cases, whereas 3.3 % had an income below $26,000 (USD). The educational levels of the participating parents were nearly identical to those of all parents of 4-year-olds in Norway. The national average and the population of Trondheim is similar on several key indicators,( e.g., the employment rate is identical to the national rate, 80.0 % of the households are two-parent families compared to the national average of 81.4 %, and the gross income per inhabitant is 99.5 % of the national average).

**Fig. 1:**
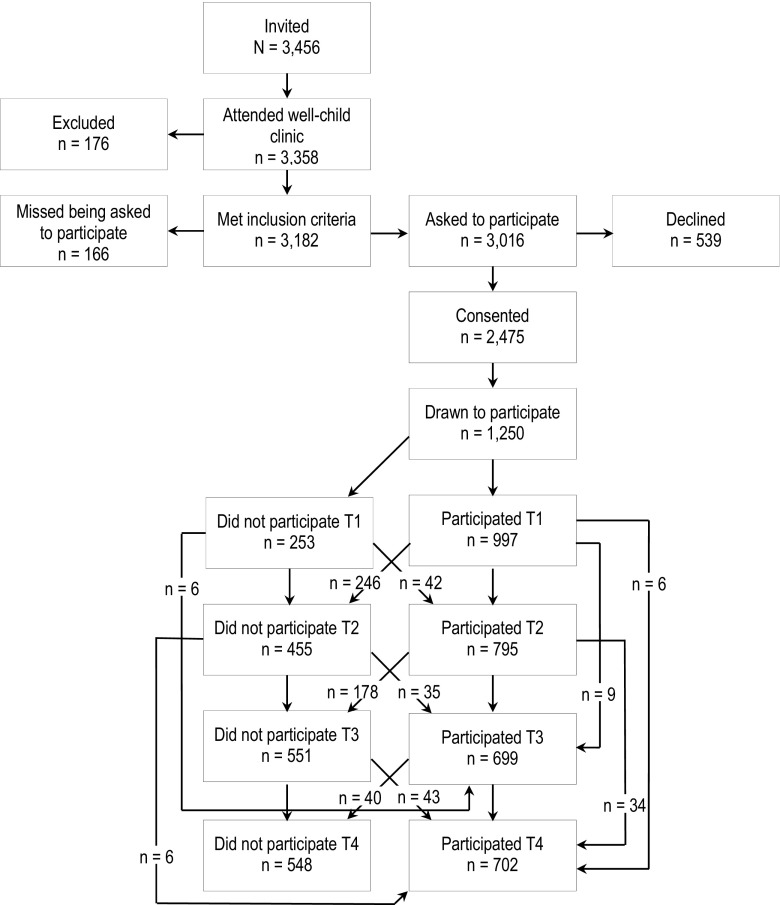
Sample recruitment and attrition

As shown in Fig. 1, almost all of the children in the two cohorts appeared at the check-up assessment; thus, the sample is effectively a community sample. Parents were informed about the present study by the health nurse using procedures approved by the Regional Committee for Medical and Health Research Ethics Mid Norway, and the health nurse obtained written consent. To increase the statistical power, we oversampled children with mental health problems by allocating the participants to four strata according to the children’s SDQ total difficulties scores (cut offs: 0–4, 5–8, 9–11, 12–40). Using a random number generator, defined proportions of consenting parents were drawn to participate. The drawing probabilities increased with increasing SDQ scores of 0.37, 0.48, 0.70, and 0.89 in the four strata, respectively. The drop-out rate after consent at the well-child clinic did not differ across the four SDQ strata, (χ^2^ = 5.70, *df* = 3, NS) or by gender (χ^2^ = 0.23, *df* = 1, NS). Mean age of the children at first assessment was 4.57 years (SD = 0.25, 49.9 % males). Retesting occurred biannually, and the mean ages were 6.72 (SD = 0.19), 8.80 (SD = 0.24), and 10.51 (SD = 0.17) years. Overall, 1042 participants had information from at least one wave of data collection and comprised the analytical sample. At age 8 and 10, attrition was higher among boys (age 8: OR = 1.37, 95 % CI:1.03–1.83; age 10: OR = 1.44, CI:1.08–1.92). Attrition was also higher at age 8 among those with more ADHD symptoms at age 4 (OR = 1.07, CI:1.00–1.14).

The clinical interviews were administered by experienced interviewers (*n* = 9) who were blinded to the SDQ scores and all other information about the family. The interviewers held at least a bachelor’s degree in relevant fields and extensive experience in working with children and families. Training in administering the interviews was provided by the group behind its development. The interviews were video-taped and tapes were randomly checked and feedback was provided to prevent interviewer and coder drift.

### Measures

#### The Preschool Age Psychiatric Assessment (PAPA) and the Child and Adolescent Psychiatric Assessment (CAPA)

The PAPA is a semi-structured psychiatric interview with parents about their children (Egger et al. 2006) and a Norwegian version approved by the creators of the PAPA was administered at age 4 and 6. In the DSM-IV, all ODD and some CD symptoms rely on normative evaluations of frequency (e.g., “often loses temper”). “Often” was defined post hoc as the highest 10 % of the population as determined by frequency counts in the present sample at the specified age (Egger et al. 2006). Six CD symptoms deemed age inappropriate are by default not included in the PAPA (i.e. “Stolen while confronting a victim”; “Forced someone into sexual activity”; “Broken into someone else’s house”; “Stays out at night”; “Run away from home overnight”; “Truant from school”). A measure of symptoms of depressive and anxiety disorders was computed as the sum of symptoms of major depressive disorder, generalized anxiety disorder, separation anxiety disorder, social anxiety disorder, and specific phobia. Blinded raters re-coded 9 % of the interview audio recordings. The interrater reliabilities using intra-class correlations were as follows: ODD = 0.97, CD = 0.91, ADHD = 0.97, and anxiety/major depressive disorder = 0.93. At ages 8 and 10 years, we applied a Norwegian version of the child and adolescent version of the PAPA, the CAPA (Angold and Costello 2000); this time, also interviewing the children. A symptom was regarded as present if it was reported by at least the child or the parent with the exception of ADHD, where only the parent was questioned (Angold and Costello 2000). The interrater reliabilities from blinded recodings of 15 % of the interviews were as follows: ODD = 0.90, CD = 0.85, ADHD = 0.90, and anxiety/major depressive disorder = 0.87*.* For both PAPA and CAPA we analyzed symptom counts of the respective disorders.

### Demographics

Parental occupation was coded according to the official Norwegian version of the International Classifications of Occupations (International Labour Office 1990; Statistics Norway 1998). Professionals and leaders were grouped together as being of high SES, whereas farmers/fishermen, skilled and unskilled workers were grouped as being of low SES. When the parents were living together, the parent with the higher-level occupation was chosen. The current marital status of the biological parents was also recorded. Those who were married or cohabitating for more than 6 months were grouped together as Cohabitating. Divorced, separated, foster parents or never married parents were grouped together as Not cohabitating.

### Statistical Analyses

Group level change and stability of symptoms were examined using latent growth curves, whereas homotypical intraindividual stability was examined using Pearson’s correlation. To examine the stability between preschool and middle childhood when adjusting for comorbidity, a multivariate regression was performed with age 10 ODD and CD symptoms as outcomes and age 4 ODD, CD, ADHD, and anxiety/depression as predictors.

To test the proposition that symptoms of ODD increase the risk of later symptoms of CD, an autoregressive cross-lagged model was constructed. Here, symptoms of ODD, CD, ADHD and anxiety/depression at one time point (t) were regressed on the same symptoms at t-1. Symptoms within each time point were allowed to correlate, as were the residuals at age 10. To account for potential sleeper effects, the effects of previous symptoms bypassing symptom levels in-between, auto-regression paths between an outcome and all previous timepoints were included. We examined the possibilities that the ODD➔CD relationship and the stability in symptoms of ODD and CD might be stronger for children with more problems at the outset by adding mean centered quadratic components to the model. Significant and positive effects would indicate that those with the highest level of symptoms had a disproportionally stronger probability of later symptoms, be it homotypical or heterotypical continuity.

Analyses were performed using Mplus version 7.31 (Muthén and Muthén 1998-2012). Missing values were handled using Full Information Maximum Likelihood Estimation (FIML) under the assumption that data were missing at random, as indicated by the attrition analyses. Due to oversampling, the results were weighted back with a factor corresponding to the number of children in the population in a particular stratum divided by the number of participants in that stratum. Because symptoms were expected to be right-skewed, a robust Maximum Likelihood estimator was used.

To examine potential gender differences, two competing models were conducted for each gender; one in which the effects from ODD to CD were fixed as similar for boys and girls, and one in which they were freely estimated. Using Wald’s test, we examined whether the fit of the two competing models differed. We applied the same procedure to test for differences in SES and marital status.

## Results

The correlations between symptom counts of all disorders at the four ages are shown in Table 1. ODD and CD were concurrently correlated in the 0.35 to 0.40 range. The correlations between ODD and ADHD and depression/anxiety were equally strong, but slightly weaker for CD.

**Table 1:** Correlations, means, SDs, range, skewness and kurtosis of the study variables

Variable	1	2	3	4	5	6	7	8	9	10	11	12	13	14
1 ODD age 4	-													
2 ODD age 6	0.29***	-												
3 ODD age 8	0.32***	0.35***	-											
4 ODD age 10	0.30***	0.32***	0.46***	-										
5 CD age 4	0.35***	0.17***	0.14**	0.19***	-									
6 CD age 6	0.25***	0.34***	0.16***	0.18***	0.24***	-								
7 CD age 8	0.13**	0.23***	0.40***	0.26***	0.17***	0.18***	-							
8 CD age 10	0.08	0.12*	0.22***	0.37***	0.17**	0.06	0.23***	-						
9 ADHD age 4	0.34***	0.23***	0.25***	0.18***	0.24***	0.12**	0.14**	0.16**	-					
10 ADHD age 6	0.20***	0.38***	0.30***	0.25***	0.16***	0.18***	0.27***	0.14**	0.41***	-				
11 ADHD age 8	0.19***	0.30***	0.35***	0.28***	0.09*	0.13**	0.29***	0.17***	0.38***	0.58***	-			
12 ANXDEP age 4	0.38***	0.26***	0.20***	0.15***	0.21***	0.11*	0.11*	0.14**	0.42***	0.17***	0.20***	-		
13 ANXDEP age 6	0.09*	0.48***	0.24***	0.21***	0.11**	0.15***	0.12**	0.10	0.20***	0.40***	0.31***	0.23***	-	
14 ANXDEP age 8	0.20***	0.28***	0.35***	0.22***	0.10*	0.13**	0.23***	0.13**	0.25***	0.32***	0.43***	0.33***	0.35***	-
M	0.67	0.96	1.07	0.77	0.30	0.22	0.30	0.23	1.05	1.30	1.20	1.60	1.72	1.83
SD	1.09	1.21	1.39	1.15	0.61	0.49	0.60	0.55	1.84	2.24	2.40	2.08	2.43	2.43
Potential range	0–8	0–8	0–8	0–8	0–9	0–9	0–15	0–15	0–18	0–18	0–18	0–32	0–32	0–32
Actual range	0–7	0–6	0–7	0–7	0–4	0–4	0–4	0–4	0–16	0–16	0–17	0–12	0–13	0–13
Skewness	2.05	1.61	1.62	2.01	2.21	2.80	2.33	3.05	3.05	2.73	2.80	1.84	1.96	1.97
Kurtosis	4.58	2.84	2.77	4.70	4.70	11.05	5.03	11.44	12.06	9.20	8.48	4.29	4.75	4.70

*ODD* oppositional defiant disorder, *CD* conduct disorder, *ADHD* attention deficit hyperactivity disorder, *Anxdep* symptoms of anxiety and depression

*p < 0.05, **p < 0.01,***p < 0.001

### The Stability of ODD and CD Symptoms from Preschool to Middle Childhood

The mean scores displayed in Table 1 indicated that there was an increase in ODD symptoms. A latent growth curve demonstrated that the slope of ODD symptoms was significant, *M*_*growth*_ = 0.02, *p* = 0.018, whereas there was no overall change in CD symptoms, *M*_*growth*_ = −0.01, *p* = 0.11. The number of ODD symptoms appeared to increase from age 4 to 8 and decline thereafter (Table 1). A model that included quadratic growth in ODD symptoms fitted the data better than a linear model, *Δχ* ^*2*^ = 62.44, *df* = 6, *p* < 0.001, and the quadratic component was negative and significant, *M*_*qgrowth*_ = −0.04, *p* < 0.001, which supports an increase and subsequent decline. No such development was observed in the CD symptoms.

As shown in Table 1, age 4 ODD predicted age 10 ODD, and such homotypical continuity was observed in CD as well, albeit weaker. When adjusting for symptoms of concurrent disorders at age 4, the homotypical continuity from age 4 to 10 remained for ODD, *B*_ODD_ = 0.26, 9 [0.13–0.37], *β* = 0.24, *p* < 0.001, and was smaller, albeit significant, for CD, *B*_CD_ = 0.13, [0.03–0.23], *β* = 0.06, *p* = 0.009.

### Predicting CD Symptoms Using Previous ODD Symptoms

**Fig. 2:**
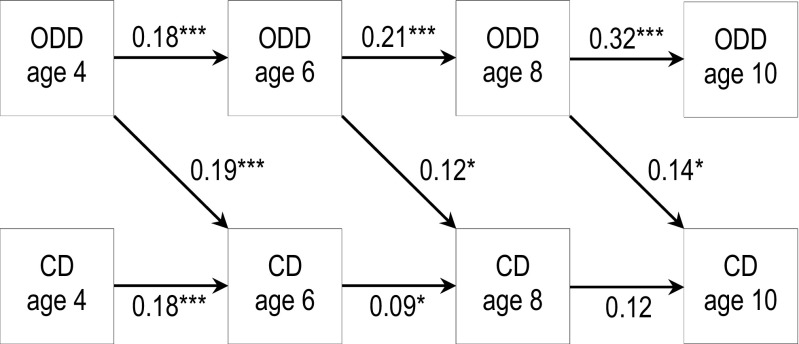
Autoregressive and cross-lagged effects between symptoms of ODD and CD from 4 to 10 years; coefficients are standardized. Symptoms of ODD and CD were also regressed on previous symptoms of ADHD, anxiety and depression. Note: * p < 0.05, *** p < 0.001. All paths are adjusted for the effects of symptoms of attention-deficit/hyperactivity disorder, anxiety and depressive disorders

Results from the autoregressive cross-lagged model are shown in Table 2; for illustrative purposes, the main results regarding the ODD➔CD relationship are depicted in Fig. 2. A good fit to the data was achieved: χ^2^ = 13.45, *df* = 18, *p* = 0.75; CFI = 1.000; TLI = 1.017; RMSEA = 0.000. More symptoms of ODD predicted more symptoms of CD at the subsequent age, even after adjusting for concurrent CD and symptoms of ADHD, depression and anxiety. It should be acknowledged that the effects were small, ranging from an increase of 0.05 to 0.09 symptoms of CD for each additional previous symptom of ODD incurred. As shown, the ODD➔CD effect was nearly identical at all ages, which was confirmed when the paths between ODD and later CD were fixed to be equal across lags; this did not deteriorate the fit (all *p*-values > 0.33). As indicated in Fig. 2, the effect of previous ODD on later CD was equally strong as the effect of previous CD on later CD. When fixing these two paths to be identical, the fit was not significantly affected at any age (6 years: Wald = 0.70, *df* = 1, *p* = 0.40, 8 years: Wald = 0.66, *df* = 1, *p* = 0.42, and 10 years: Wald = 1.70, *df* = 1, *p* = 0.19). As shown in Table 2, CD at age 8 was also predicted by ADHD at age 6, but not at the ages before or after. ODD at ages 6 and 8 was predicted by previous ADHD, and this effect bordered on significance at age 10. Anxiety and depression at age 4 did predict ODD at age 6, but not at later ages. The effects of previous ODD on CD two years later did not differ according to gender, SES or cohabitating status at any time point. Moreover, no differences in ODD and CD stability were found based on gender, SES or cohabitating status. Finally, no quadratic effects emerged with respect to the ODD➔CD relationship or the homotypical stability of ODD and CD.

**Table 2:** Prediction of ODD and CD symptoms from previous symptom levels, 4 to 10 years

	ODD			CD		
	B	95 % CI	p	B	95 % CI	p
	Age 6					
ODD – age 4	0.20	0.10–0.30	<0.001	0.09	0.04–0.13	<0.001
CD – age 4	0.13	-0.02–0.28	0.092	0.14	0.07–0.21	<0.001
ADHD – age 4	0.07	0.02–0.12	0.004	0.01	-0.02–0.03	0.65
ANXDEP – age 4	0.09	0.03–0.14	0.001	0.00	-0.02–0.02	0.98
R^2^	0.13			0.10		
	Age 8					
ODD – age 6	0.25	0.13–0.36	<0.001	0.06	0.03–0.11	0.013
CD – age 6	0.04	-0.17–0.26	0.73	0.12	0.00–0.23	0.050
ADHD – age 6	0.10	0.05–0.16	0.001	0.06	0.03–0.10	0.000
ANXDEP – age 4	0.30	-0.03–0.08	0.32	-0.01	-0.04–0.01	0.21
ODD – age 4	0.24	0.12–0.36	<0.001	-0.01	-0.06–0.04	0.024
CD – age 4	0.03	-0.15–0.20	0.74	0.10	0.01–0.18	0.82
R^2^	0.21			0.13		
	Age 10					
ODD – age 8	0.26	0.16–0.36	<0.001	0.05	0.01–0.10	0.032
CD – age 8	0.07	-0.14–0.29	0.49	0.11	-0.01–0.24	0.076
ADHD – age 8	0.04	-0.01–0.09	0.071	0.02	-0.01–0.04	0.14
ANXDEP – age 8	0.01	-0.05–0.06	0.89	0.01	-0.01–0.03	0.43
ODD – age 4	0.13	0.03–0.22	0.014	-0.02	-0.06–0.02	0.38
CD – age 4	0.10	-0.06–0.20	0.20	0.12	0.03–0.22	0.012
ODD – age 6	0.12	0.03–0.20	0.045	0.00	-0.05–0.05	0.99
CD – age 6	0.08	-0.13–0.29	0.44	-0.03	-0.12–0.07	0.54
R^2^	0.28			0.10		

*ODD* oppositional defiant disorder, *CD* conduct disorder, *ADHD* attention deficit hyperactivity disorder, *Anxdep* symptoms of anxiety and depression

## Discussion

We examined whether symptoms of ODD assessed with structured clinical interviews predicted symptoms of CD two years later in the age span from 4 to 10 years in a large community sample. The results support our hypothesis that symptoms of ODD increase the risk of later symptoms of CD in early and middle childhood, as symptoms of ODD predicted symptoms of CD between the ages 4 to 6, 6 to 8 and 8 to 10 years. Moreover, as expected, there was modest stability in symptoms of ODD and CD from age 4 to 10, even after adjusting for comorbidity.

### The Effect of ODD on Symptoms of Early Onset CD

In a range of previous research on both older and younger children, ODD has often disappeared as a predictor of CD when previous symptoms of CD are adjusted for (Diamantopoulou et al. 2011; Rolon-Arroyo et al. 2014), or this effect has been significantly reduced (Lahey et al. 2009). Moreover, in older children and adolescents, the predictive power of ODD symptoms typically vanishes when controlling for co-occurring symptoms of ADHD, depression and anxiety (Costello et al. 2003). However, as for symptoms of early onset CD, we found that ODD symptoms remained a significant predictor of later CD symptoms even after controlling for previous CD symptoms and comorbid conditions. These results are consistent with some questionnaire studies of younger children (e.g., Drabick et al. 2011), and they are also consistent with some findings among referred children (Burke and Loeber 2010). Our results add to our knowledge base by demonstrating this relationship repeatedly over several time points and in a community sample.

Why do we find ODD symptoms to increase the risk of early emerging symptoms of CD whereas others do not? We have no simple explanation for this finding; nevertheless, we draw attention to two possibilities. First, by using clinical interviewer-based interviews, which arguably better identify ODD and CD than rating scales, we may have been advantageously positioned to estimate the relationship between ODD and CD, a relationship that may be less clear with less valid measures. Second, when effects of previous ODD on later CD have been detected, the magnitude of these effects has always been small (Drabick et al. 2011; Lahey et al. 2009), as was the case in the present study. Using continuous measures of ODD and CD and involving a large sample, we obtained increased power to detect these ODD➔CD effects.

The rates of ODD and CD are lower in Scandinavia than many other parts of the world, including the US (Heiervang et al. 2007; Wichstrøm et al. 2012). This lower prevalence implies that there should be fewer deviant peers with which to affiliate. This supposition is significant because associating with deviant peers may be one of the forces driving the escalation of behavioral problems (Eivers et al. 2012; Moffitt 1993). The lesser opportunity to find such peers may therefore reduce stability as well as the strength of the ODD➔CD transition in Norway as compared to countries with higher rates of problems. A strong effect of socialization with deviant peers on anti-social behavior has most often been found in late childhood and adolescence (Gifford-Smith et al. 2005; Moffitt 1993). Although there is some evidence for a concurrent or short-lived effect of affiliating with antisocial peers on antisocial behavior in young children, (i.e preschoolers), there is limited evidence for a prospective effect on change in antisocial behavior in this age group, possibly due to the unstable nature of younger children’s friendships (Eivers et al. 2012). Thus, finding a consistent ODD➔CD relationship from early childhood to middle childhood, even in the context of low prevalence of symptoms and disorder, might attest to the validity of our findings. Nevertheless, future research in countries where disorders are more common should investigate whether the ODD➔CD relationship during early and middle childhood differs from that identified in the current study.

### The Stability of ODD and CD Symptoms from Preschool to Middle Childhood

Studying clinical samples (Bunte et al. 2014; Keenan et al. 2011; Speltz et al. 1999), investigators have reported fairly high stability coefficients for ODD and CD,( i.e., approximately half or more of the sample show the same disorder at follow-up), with reported odds ratios ranging from 3.2 to 22.6. However, findings from such samples may not accurately portray the stability in the population because the stability may simply be different for children without any major initial problems, which is the case for most children in community samples, due to a variety of reasons, including regression towards the mean, treatment and the effect of differing comorbidity. When community samples have been studied starting in preschool, parent- or teacher-completed rating scales have almost always been used. Findings from such studies also indicate fairly high short-term and longer-term stability in symptoms of ODD and CD (Diamantopoulou et al. 2011; Drabick et al. 2011; Kim-Cohen et al. 2005, 2009). However, using a structured interviewer-based diagnostic interview in a community sample, we found lower short- (i.e., in the 0.33 to 0.47 range) and longer-term (i.e., 0.30) stability coefficients for ODD, and even lower values for CD (short-term 0.18 to 0.24 and longer-term 0.17). This finding is consistent with the results obtained from a shorter-term longitudinal study employing a diagnostic interview, which only found a moderate stability of ODD (Lavigne et al. 2001). However, another shorter-term study using a diagnostic interview reported higher stability (Bufferd et al. 2012). One explanation for the higher stability when rating scales are used might be that these scales overestimate stability in young children’s symptoms because they not only capture behavioral stability but also the stability of the reporter’s (most often a parent) perspective of the child.

It should also be acknowledged that the rather small size of the autoregressive coefficients along with consistent sleeper effects from previous lags and fairly stable symptom means indicate considerable waxing and waning of symptoms over time, particularly with regards to CD. Thus, although symptoms of ODD and CD may diminish or even vanish in the short term, the present constellation of results indicates that for some children, these symptoms might reappear later in childhood.

### Limitations

Some limitations should be acknowledged. First, we examined symptom counts, not disorders. We do not know whether the identified ODD➔CD relationships and stabilities would be different if diagnoses were examined. This examination would require even larger samples or populations where the rates of these disorders are higher than those in Scandinavia (Heiervang et al. 2007; Wichstrøm et al. 2012). No quadratic effects were observed, however, which suggest that the homotypical stability and the effect of ODD on later CD were the same across all levels of symptoms. These results provide initial support for the hypothesis that the present findings should hold for subclinical and clinical levels of ODD and CD. Second, the ODD ➔CD effect was small. Even so, a modest reduction in the probability of additional symptoms of CD emerging could prevent a substantial amount of children from crossing the diagnostic threshold, at the population level. This possibility of prevention in the face of subclinical ODD concurs with the findings of Keenan et al. (2011); a substantial share of the preschoolers in their sample who met DSM-IV criteria for CD at follow-up had 1–3 symptoms of ODD at baseline. Third, although the sample in this study was representative of the Norwegian population and because it was relatively homogenous with regard to SES and ethnicity, it likely differs in relevant aspects from other populations (e.g., the US, where much of the ODD and CD literature has originated). Moreover, the absolute level of the disorders and their symptoms are lower in Norway compared with most other countries (Heiervang et al. 2007; Wichstrøm et al. 2012), including the US (Nock et al. 2006, 2007), Spain (Ezpeleta et al. 2014) and Brazil (Murray et al. 2013; Petresco et al. 2014). Differing rates of potential etiological factors on child, family and society levels might help explain this seemingly “Nordic advantage” (Heiervang et al. 2008). Unsurprisingly, the prevalence of externalizing behavioral disorders are higher among children from low-SES families and those living under adverse environmental conditions in any country (Huaqing Qi and Kaiser 2003). As such, we examined the heterotypical and homotypical continuity among children from low-SES families and children of divorce; we found no differences compared with the rest of the sample. Thus, although the absolute level of the disorders and symptoms might differ among groups and countries, these results partially suggest that the mechanisms at play are similar across groups. In accordance with a “common mechanism view”, treatment studies of ODD and CD (e.g. The Incredible Years programs, Reid and Webster-Stratton 2001) have shown comparable effects in Norway with those originating from American and Anglo-Saxon countries (Larsson et al. 2009). These treatments exert their effects via the same mechanisms (e.g., reduction of harsh parenting, enhancement of positive parenting and perceived parenting competence) as those reported from studies in other cultures (Reedtz et al. 2011). Again, these findings indicate that although cross-cultural differences might be found in the absolute levels of disorders, the etiology of symptom maintenance or reduction might be similar in countries with either high or low rates. Fourth, the present inquiry did not examine the potential mechanisms through which early ODD results in early onset CD; rather, it described the ODD➔CD relationship, its stability and its continuity. The available literature on the onset and maintenance of ODD and CD symptoms offers a range of mechanisms that might contribute to the progression from ODD to CD such as the individual differences found in children, including neurochemical or genetic factors, family factors, and neighborhood factors (Holmes et al. 2001). After the ODD➔early onset CD effect in younger children is soundly established, future studies should address whether suggested mechanisms for older children (Holmes et al. 2001) also operate on this relationship at both subclinical and clinical levels. Finally, although we used the FIML method to correct for selective attrition, attrition might have been selective according to non-measured factors that could have affected the results.

In conclusion, the present study shows that symptoms of ODD increase the risk of subsequent symptoms of CD throughout the period from preschool to middle childhood. Indeed, the contribution of previous ODD to future CD symptoms is as strong as for previous CD symptoms. The prevalence of ODD symptoms increases from preschool to 8 years of age and declines thereafter. Both ODD and CD symptoms show some homotypical continuity during this period and are stronger for ODD than CD. This continuity in symptoms, combined with an increased risk of early symptoms of CD forecasted by symptoms of ODD, underscores the importance of detection, prevention and treatment of behavioral disorders already in early childhood.

## Abbreviations

ODD: oppositional defiant disorder
CD: conduct disorder
